# Genome Sequence of the Basidiomycetous Fungus *Pseudozyma aphidis* DSM70725, an Efficient Producer of Biosurfactant Mannosylerythritol Lipids

**DOI:** 10.1128/genomeA.00053-14

**Published:** 2014-02-13

**Authors:** Stefan Lorenz, Michael Guenther, Christian Grumaz, Steffen Rupp, Susanne Zibek, Kai Sohn

**Affiliations:** a Fraunhofer IGB, Stuttgart, Germany; b University of Stuttgart IGVP, Stuttgart, Germany

## Abstract

*Pseudozyma aphidis* is an efficient producer of mannosylerythritol lipids exceeding concentrations of >100 g/liter from renewable feed stocks. Additionally, a biosurfactant cellobiose lipid is also secreted during nitrogen limitation. Here, we describe the sequencing of *P. aphidis* to unravel the genomic basis of biosurfactant metabolism in *P. aphidis*.

## GENOME ANNOUNCEMENT

Mannosylerythritol lipids (MEL) belong to the most promising microbial biosurfactants and are secreted by fungi of the genera *Pseudozyma* and *Ustilago*, of which *Pseudozyma aphidis* facilitates product concentrations of up to 165 g/liter. This species secretes a mixture of the MEL-A, -B, -C, and –D, which share a common sugar group, two fatty acid residues of medium chain length, and different numbers of acetyl groups. In *Ustilago maydis*, the gene cluster for MEL biosynthesis encodes the glycosyltransferase Emt1p, two acyltransferases Mac1p and Mac2p, as well as a transporter protein Mmf1p and the acetyltransferase Mat1p (1). A homologous cluster was found in *Pseudozyma antarctica* T-34 (2) and *Pseudozyma hubeiensis* (3), indicative of a conserved biosurfactant metabolism. However, *P. antarctica* T-34 and *P. aphidis* revealed significant differences in substrate-dependent induction of MEL synthesis compared to that of *U. maydis* (4). Beyond that, *P. aphidis* secretes an additional cellobiose glycolipid.

Here, we describe the draft genome sequence of the MEL-producing species *P. aphidis* DSM70725. For this purpose, we sequenced the corresponding genomic DNA to approximately 90-fold coverage using the Illumina platform (HiSeq 2000), comprising a total amount of 35,141,960 reads, each 50 nucleotides in length. In addition, we also sequenced a paired-end cDNA library of the *P. aphidis* transcriptome comprising 48,195,420 read pairs with 2 × 95 nucleotides in length and approximately 300 nucleotides insert size (HiSeq 2000). Running the Velvet short-read assembler (5) using the genomic fragments generated an initial assembly of 2,160 contigs. Expanding these contigs by SSPACE (6) and the additional reads from cDNA sequencing reduced the total number to 1,968 contigs, resulting in 17.92 Mb for the whole genome of *P. aphidis* (longest contig, 78.1 kb; shortest contig, 1.05 kb; N_50_, 14.7 kb), revealing a G+C content of 61.2%. In a next step, we blasted (BLASTn) these nucleotide contigs against the genome of the closely related species *P. antarctica* T34. The total alignment length of the top BLAST hits was 14.1 Mb for 1,950 contigs, with an average identity of 97.63%. This alignment permitted an assignment to 24 supercontigs, whereas 18 remaining contigs could not be aligned. For the annotation of newly *in silico*-predicted genes, we applied Augustus (7), using *U. maydis* as a reference species. Accordingly, we detected 6,011 potential complete protein-coding sequences, with an average length of 1,875 nucleotides (longest coding sequence [CDS], 16,854 nucleotides [nt]; shortest CDS, 198 nt). Searching this open reading frame (ORF) collection for homologs in the nr database of NCBI revealed 5,589 hits, with an average identity of 74.51% of the top BLAST hits (BLASTp E value, <1E - 10). Strikingly, we identified the complete MEL biosynthesis gene cluster at the beginning of supercontig 20, which included *EMT1*, *MAC1*, *MAC2*, *MMF1*, and *MAT1*. All five relevant *MEL* genes are significantly conserved between *P. antarctica* T34 and *P. aphidis*, with similarities of 89.4%, 86.8%, 91.2%, 87%, and 86.8% at the nucleotide level, respectively. These results indicate that a similar MEL pathway exists in *P. aphidis* as was already shown for *P. antarctica* and *U. maydis* (1, 2, 8).

### Nucleotide sequence accession numbers.

This whole-genome shotgun project has been deposited at DDBJ/EMBL/GenBank under the accession no. AWNI00000000. The version described in this paper is version AWNI01000000.
